# Extending the straight leg raise test for improved clinical evaluation of sciatica: validity and diagnostic performance with reference to the magnetic resonance imaging

**DOI:** 10.1186/s12891-021-04649-z

**Published:** 2021-09-21

**Authors:** Janne Pesonen, Michael Shacklock, Juha-Sampo Suomalainen, Lauri Karttunen, Jussi Mäki, Olavi Airaksinen, Marinko Rade

**Affiliations:** 1 Department of Rehabilitation, Kuopio University Hospital, PL 100, 70029 KYS / Kuopio, Finland; 2 Department of Surgery (incl. Physiatry), University of Eastern Finland, Kuopio, Finland; 3 Neurodynamic Solutions, Adelaide, Australia; 4 Department of Clinical Radiology, Kuopio University Hospital, Kuopio, Finland; 5 Faculty of Medicine, University of Osijek, Orthopaedic and Rehabilitation Hospital “Martin Horvat”, Rovinj, Croatia; 6 Department of Natural and Health Studies, Juraj Dobrila University of Pula, Pula, Croatia

## Abstract

**Background:**

The straight leg raise test (SLR) is one of the most utilized and studied physical tests in patients with low back pain (LBP) for the detection of lumbar disc herniation (LDH), showing high sensitivity and heterogeneous or low specificity. The high incidence of asymptomatic ‘pathologic’ findings in the magnetic resonance imaging (MRI) scans may cause verification bias to these results. We studied an extended SLR (ESLR) by adding location-specific structural differentiation movements (hip internal rotation or ankle dorsiflexion) to the traditional SLR for it to better differentiate neural symptoms from musculoskeletal. Previously, the ESLR has shown almost perfect interrater reliability between examiners and ability to detect sciatic patients. In this study, we investigated whether a ’positive’ ESLR finding is associated with pathology seen on MRI.

**Methods:**

Forty subjects comprised the study population, 20 in sciatic group and 20 in control group. The ESLR was performed ‘blinded’ to the subjects. After the ESLR, each subject’s lumbar MRI was evaluated. The MRIs were analyzed independently by 2 senior radiologists and a spine specialist clinician. The ESLR and MRI results were cross-tabulated. To obtain the odds ratio (OR) with positive ESLR or SLR results for LDH or nerve root compression (NC), a binary logistic regression analysis with subjects’ age, gender, height and weight was performed. ESLR’s validity was assessed by combination of interrater agreement and percentage prevalence of both LDH and NC.

**Results:**

Of sciatic (ESLR+) patients, 85 % had LDH and 75 % NC in the MRI. Not surprisingly, MRI showed a very high incidence of ‘false-positive’ findings with the ESLR negative group. The ESLR showed 0.85 sensitivity and 0.45 specificity for LDH and 0.75 sensitivity and 0.50 specificity for NC. A positive result in the ESLR was found to be strongly associated with for both LDH and NC: the OR was 8.0 (*p* = 0.028) and 5.6 (*p* = 0.041), respectively.

**Conclusions:**

The ESLR shows high validity in detecting neural symptoms and is strongly associated with pathology seen in the MRI when judged positive. We suggest the use of ESLR in clinical practice as a part of clinical examination, where it may prove to be a valuable tool in detecting patients with sciatic symptoms.

## Background

Low back pain is one of the main causes of disability [1] and leads to a marked socioeconomic burden worldwide [2]. Despite numerous efforts, its incidence has increased generating high demand on healthcare systems for resources and effective treatment [3]. Needed at all levels of medicine are reliable and accurate measures to identify and discern different low back pain subtypes in this large patient group [4].

In recent decades, magnetic resonance imaging (MRI) has become both widely available and employed in an attempt to form an accurate pathoanatomical diagnosis causing low back pain [2]. It is conspicuously utilized in the presence of pain that radiates into the lower extremities, usually named as sciatica, to determine the presence of lumbar disc herniation and nerve root compression. As the MRI itself is highly sensitive and known to show a high prevalence of asymptomatic findings in the spine [5], these findings can easily be presumed to cause a patient’s symptoms and, consequentially, may lead to invasive and expensive treatments [3, 4, 6, 7]. Following this, there is a recognized need for tools in back pain assessment that meet patient satisfaction and increase clinician’s knowledge while avoiding unnecessary imaging [8].

The straight leg raise test (SLR) is one of the most utilized physical tests in patients with low back pain [9, 10]. It has been studied intensively for the detection of lumbar disc herniation for which it shows high sensitivity and heterogeneous or low specificity [11, 12]. In most of these studies, MRI has been the reference standard. Due to the high incidence of asymptomatic “pathological” findings seen in the MRI scans [5], it can be debated that the results may be susceptible to verification bias [13]. To address this issue, and also to provide a cost-effective and reliable way to evaluate low back pain patients especially with radicular symptoms, we modified the traditional SLR by adding location-specific structural differentiation movements (hip internal rotation or ankle dorsiflexion) to increase the traditional SLR’s ability to detect neural symptoms from those of musculoskeletal origin, hence the name extended SLR (ESLR). The ESLR has already shown both almost perfect interrater reliability between examiners and ability to detect sciatic patients, even when utilized alone without any knowledge of patient history or other clinical data [14].

In this present study, we followed the same patient group as in the ESLR reliability study [14] and determined the prevalence of MRI findings with both sciatic and control subjects. We also investigated whether the ESLR test result was associated with (pathological) findings seen on the MRI, which in turn may provide help in the clinical decision-making.

## Methods

The institutional ethical committee approved all aspects of this study that involved human subjects. All subjects signed an informed consent form to participate in the study under the Declaration of Helsinki.

### Study population

The study population consisted of the same 40 subjects that participated in the ESLR interrater reliability study [14], 20 in the sciatic symptomatic group and 20 in the control group including nonspecific low back pain patients and hip pain patients. These subjects were gathered from consecutive institutional spine center patients willing to participate in the study in order of appearance to the center. First, the study controller took a complete patient history and performed an in-depth clinical examination including neurological examination of the lower leg and ESLR. The subject allocation to groups was made by the study controller based on the combination patient history, symptoms and clinical findings which have been shown to be reliable in detecting sciatic patients [11, 15]. A complete list of inclusion and exclusion criteria is shown in Table 1. After group allocation, the interrater reliability test part for ESLR was performed by 2 independent blinded examiners (both physiatry residents) [14]. After the ESLR, each subject’s MRI of the lumbar spine was evaluated. The results of the traditional SLR (negative/positive) were retrieved from the patient’s medical records performed by the treating physician.

**Table 1:** Exclusion and inclusion criteria

**Exclusion criteria:**
∙ Known spinal tumor or malignacy
∙ Incomplete and/or painful knee extension
∙ Previously known other joint involvement, such as rheumatoid arthritis or already recognized metabolic bone disease
∙ Age more than 65 years or younger than 18 years
∙ Subjects' refusal to give informed consent to participate
∙ Claustrophobia on unwillingness to undergo magnetic resonance imaging
**Sciatic group - Inclusion criteria:**
∙ A combination of sciatic symptoms and clinical findings indicative of sciatica
∙ A 'positive' ESLR at clinical examination by study controller
∙ Radiating pain to lower limb either below or above the knee
**Control group - Inclusion criteria:**
∙ Local low back pain, greater trochanteric/hip/groin pain, with or without hamstring tightness
∙ No signs of sciatica in clinical examinitation
∘ ESLR 'negative' performed by the study controller with no neurological findings indicating radiculopathy

ESLR = Extended straight leg raise test

The size of the study population was determined following the recommendations for reliability studies in clinical sciences where the sample size of 40 was required for the Kappa statistic to be significantly greater than 0.40 (assuming 80 % power and 0.05 significance) [16, 17], where 0.40 represents the value of the null hypothesis. In our previous study, the interrater agreement for the result of the ESLR has been shown to be almost perfect agreement between the blinded examiners as measured with Cohen’s Kappa method (0.85, *p* < 0.001) [14].

### ESLR procedure and interpretation

**Fig. 1:**
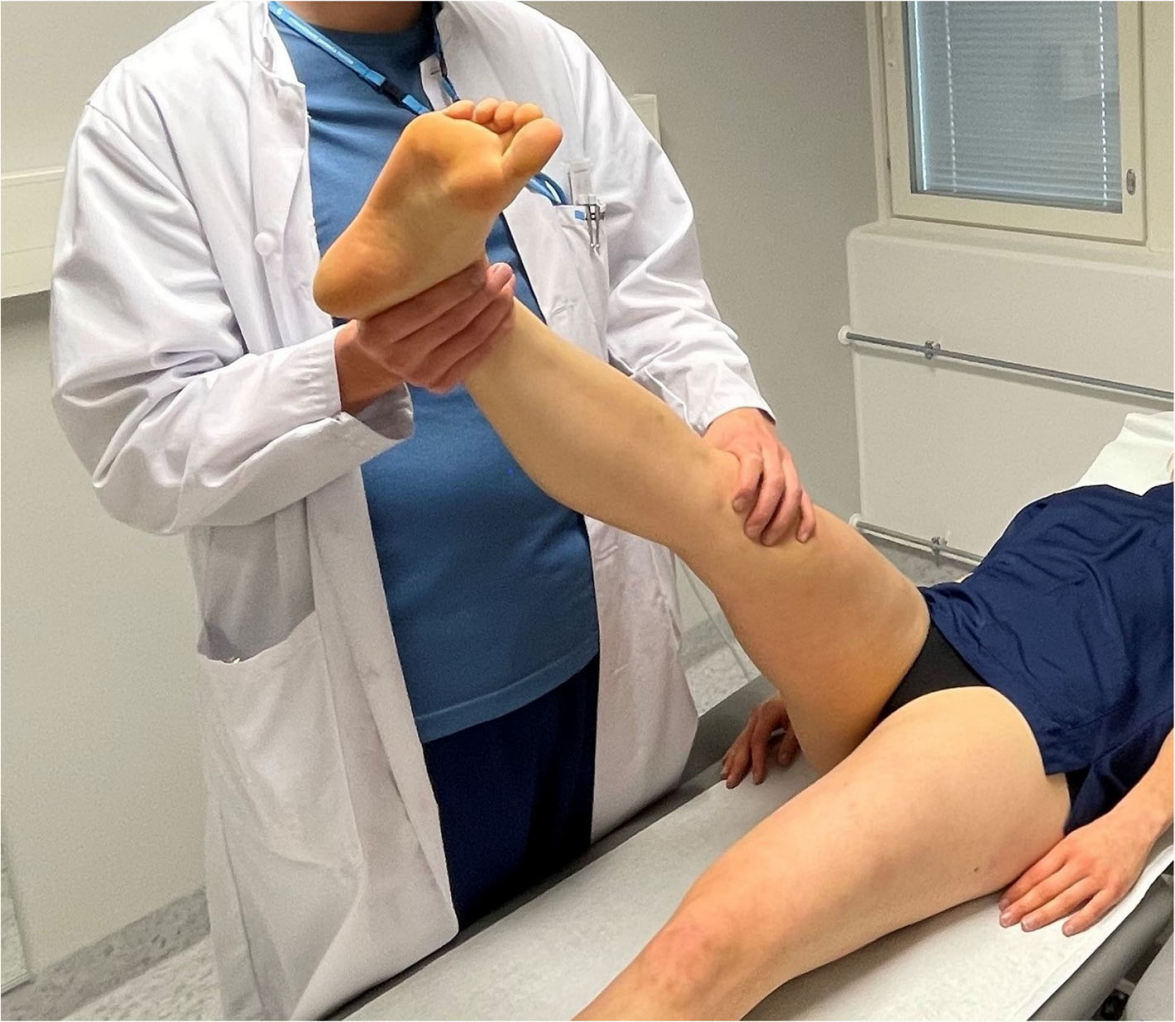
Proximal structural differentiation for distal symptoms with hip internal rotation. Published earlier by Pesonen et al., BMC Musculoskeletal Disorders 2021 [14]

**Fig. 2:**
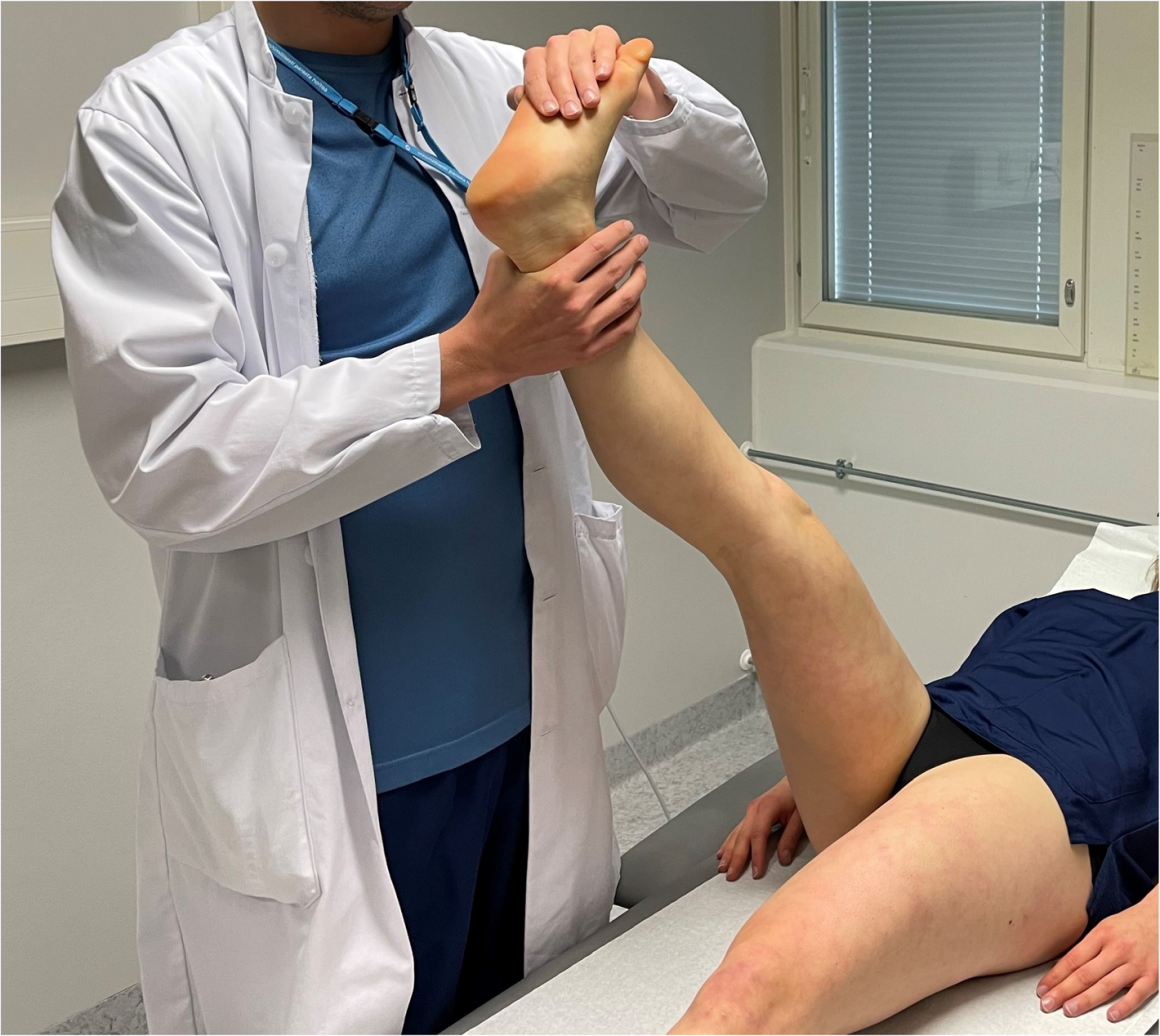
Distal structural differentiation for proximal symptoms with ankle dorsiflexion (also known as Bragard test). Published earlier by Pesonen et al., BMC Musculoskeletal Disorders 2021 [14]

The ESLR started similar to the traditional SLR [11]: the patient lies supine on the examination table and the examiner passively lifts the subject’s leg with knee straight (fully extended), hip in neutral rotational position and ankle hanging free. The leg raise was continued until the first symptoms were evoked or the subject’s ongoing symptoms in the lower extremity are aggravated by 30 %. If no responses are evoked by the hip flexion angle reaches 90 degrees, the leg raise is ceased, and the test is considered negative. The ESLR differs from the traditional SLR as the symptoms emerge: (i) the evoked responses do not need to reach below the knee, (ii) these responses do not need to emerge before 70 degrees but can happen anywhere from 0 to 90 degrees of hip flexion, and (iii) a structural differentiation maneuver is added to the SLR. To evaluate whether the evoked responses are neural or musculoskeletal in origin, a location-specific structural differentiation movement is added to the test at the hip flexion angle where these responses have been evoked/provoked. The added movement is selected based on the location of the evoked responses: If the symptoms are located distally, below the knee, a hip internal rotation (Fig. 1) is performed at the same hip flexion angle of evoked responses. In case the symptoms are provoked proximally in the buttock and/or hamstring area, ankle dorsiflexion (Fig. 2) is the differentiating movement similar to the Bragard test [18]. An integral part in performing these structural differentiation movements is that, given the structural continuum of the nervous system, a movement known to move the sciatic neural structures and its contiguous nerve roots [19–21] at an asymptomatic musculoskeletal location may evoke referred responses in the area of interest.

The ESLR was deemed positive if: (i) at least part of the patient’s symptoms were evoked (or present symptoms increased by 30 %) with the traditional SLR, and (ii) a differentiating movement that moves the nerve roots - but not the spine - increased the evoked symptoms, e.g. ankle dorsiflexion or hip internal rotation.

### MRI procedure and classification

Each subject’s MRI was performed with a 1.5 T MRI device (Siemens Magnetom Aera, Erlangen, Germany). The imaging area was from L2 level to S3 level and centered to L4-L5 disc. T1- (repetition time (TR) 500ms, echo time (TE) 11ms, 20 slices, slice thickness (ST) 3mm, field of view (FOV) 300mm, in-plane resolution 0.8 × 0.8mm, flip angle (FA) 150°), T2-weighted sagittal sequences (TR 4000ms, TE 95ms, 20 slices, ST 3mm, FOV 300mm, in-plane resolution 0.8 × 0.8mm, FA 150°) and T2-axial images (TR 1500, TE 119ms, 80 slices, ST 2mm, FOV 200mm, in-plane resolution 0.8 × 0.8mm, FA 150°) were taken to detect the presence of nerve root compression and/or lumbar disc herniation in L4/5 and L5/S1 discs. The images were viewed and analysed with Sectra PACS workstation (Sectra Workstation IDS7, version 21.2.5.6173–2019 – Sectra AB, Sweden).

**Fig. 3:**
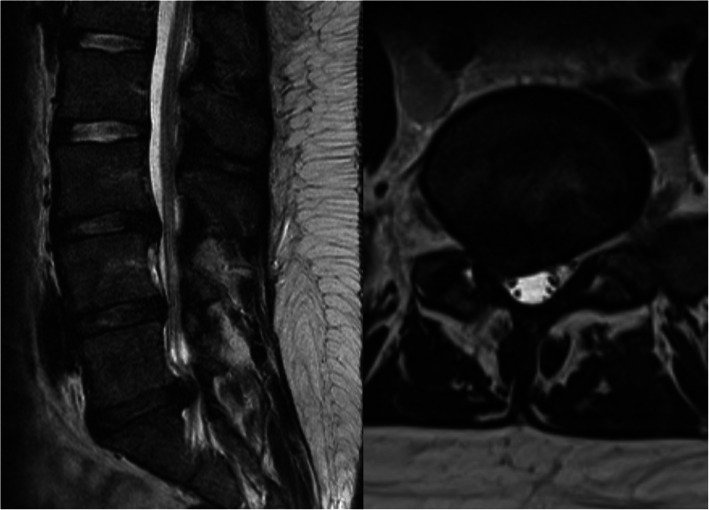
MRI showing lumbar disc herniation on L5-S1 disc with neural compression on right S1 nerve root (T2-weighted sagittal and axial views)

The MRI results were classified following the recommendations by Li et al. [22] For nerve root compression we used a 5-point scaling by van Rijn et al. [23], where groups 4 and 5 (i.e., possible root compression and definitely root compression, respectively) were judged as ‘nerve root compression positive’. We used the Combined Task Force classification as described by Fardon et al. [24] for the definition of lumbar disc herniation where extrusion, herniation or sequestration was required to be visible in the L4/L5 or L5/S1 discs on the subjects’ MRI scans (Fig. 3). The MRI images were analyzed independently and blinded from the patient’s clinical findings by 2 senior radiologists and a spine specialist clinician. From these results, a consensus for the lumbar disc herniation and nerve root compression results was formed. If there was a difference in the initial outcome (lumbar disc herniation or nerve root compression), the scans were analyzed together to form a conclusion for the outcome.

### Outcome measures and statistical analysis

The data were analyzed using IBM SPSS Statistics version 26 and Microsoft Excel for Office365 programs. Demographic variables were expressed as means with standard deviations. The prevalence of lumbar disc herniation and nerve root compression was calculated within the study groups. The outcomes of ESLR and SLR were compared with the MRI results and cross-tabulated from which the according sensitivity and specificity values were calculated. Cohen’s Kappa statistic was used to assess the agreement between the MRI and both the ESLR and SLR results. 95% confidence intervals (95%CI) were calculated. To assess the association between the ESLR or SLR results and (pathological) findings on MRI, odds ratio (OR) was calculated for lumbar disc herniation or nerve root compression using a binary logistic regression analysis adjusted with subjects’ age, gender, height and weight was performed. *P*-values < 0.05 were set to indicate statistically significant results. ESLR test’s validity was assessed for its positive result by combination of interrater agreement (Cohen’s Kappa) and percentage prevalence of both lumbar disc herniation and nerve root compression.

## Results

Forty subjects constituted the study population, 25 women and 15 men. Mean age was 41±14 years, height 170±9 cm and weight 80±22 kg. In our subject sample, a total of 28 lumbar disc herniations and 25 neural compressions were visible in the subject’s MRI scans. All subjects in the control group were evaluated as negative with both ESLR and the traditional SLRs. However, with the sciatic patients, 10/20 were judged negative with the traditional SLR due to either hip flexion angle reaching over 70 degrees when the test evoked responses to the subject (6 subjects) or the evoked symptoms did not reach below (distal) to the knee (4 subjects).

**Fig. 4:**
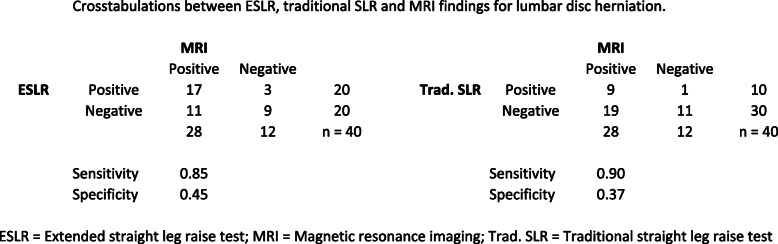
Crosstabulations between ESLR, traditional SLR and MRI findings for lumbar disc herniation. ESLR = Extended straight leg raise test; MRI = Magnetic resonance imaging; Trad. SLR = Traditional straight leg raise test

**Fig. 5:**
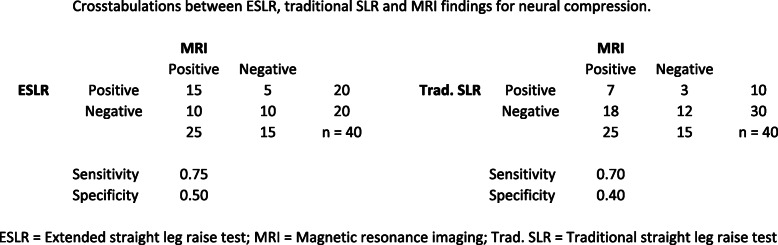
Crosstabulations between ESLR, traditional SLR and MRI findings for neural compression. ESLR = Extended straight leg raise test; MRI = Magnetic resonance imaging; Trad. SLR = Traditional straight leg raise test

Among the ESLR + subjects, 17/20 (85 %) had lumbar disc herniation and 15/20 (75 %) nerve root compression in the MRI scans. Not surprisingly, there was a very high incidence of “false-positive findings” in the MRI with the ESLR negative group: 11/20 had lumbar disc herniation and 10/20 showed nerve root compression without clinical signs of sciatic radiculopathy. In comparison, among the traditional SLR + subjects 9/10 (90 %) had lumbar disc herniation and 7/10 (70 %) nerve root compression visible in the MRI, whereas in SLR- 19/30 (63 %) and 18/30 (60 %) showed “false-positive” lumbar disc herniation and nerve root compression findings in the MRI, respectively. Cross-tabulations with sensitivity and specificity values can be found in Figs. 4 and 5.

**Fig. 6:**
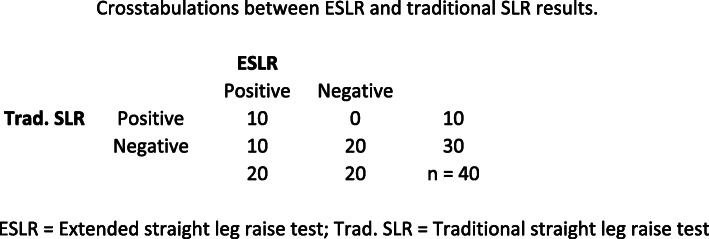
Crosstabulations between ESLR and traditional SLR results. ESLR = Extended straight leg raise test, Trad. SLR = Traditional straight leg raise test

A cross-tabulation between the ESLR and traditional SLR is shown in Fig. 6. The Cohen’s Kappa values for agreement between the findings in the MRI and the ESLR or traditional SLR are presented in Table 2.

A positive result in the ESLR was strongly associated with both lumbar disc herniation and neural compression as OR for lumbar disc herniation was 8.0 (*p* = 0.028, 95 %CI 1.3–51.2) and 5.6 (*p* = 0.041, 95 %CI 1.1–29.0) for neural compression, both statistically significant. On the other hand, the ORs for traditional SLR result did not reach statistical significancy with neither lumbar disc herniation 8.3 (*p* = 0.11, 95 %CI 0.6–109.4) nor nerve root compression 2.4 (*p* = 0.34, 95 %CI 0.4–13.8).

## Discussion

In the present study, we found that a positive result with the ESLR shows high validity and is strongly associated with both lumbar disc herniation and nerve root compression seen in the MRI. On the other hand, there was also a very high incidence of ‘false positive’ findings in the MRI in the control group. This discrepancy leads to poor overall agreement between the MRI and ESLR or traditional SLR results.

In the recent literature, there is a well-recognized need for measures to better discern the different subtypes of low back pain [4]. In this study, we analyzed the diagnostic performance of the ESLR, a variation of a widely used and known SLR test to fulfill this need. The ESLR was found to be effective in discerning the subjects with sciatic neural symptoms from those in the control group (hip pain or low back pain without sciatica). When the ESLR results were compared to the MRI results, 17 out of 20 subjects in the ESLR+ (sciatic) group showed a lumbar disc herniation and 15/20 demonstrated nerve root compression visible in the MRI scans. This translated to an 8-fold risk for lumbar disc herniation and a 5.6-fold risk for nerve root compression with a positive ESLR, both statistically significant. Even though the OR for detecting lumbar disc herniation may seem comparable between ESLR and traditional SLR (8.3 but statistically not significant), the traditional SLR could pick only 10 of the 20 sciatic subjects in our tested population. Moving to the nerve root compression findings, the differences in detection capacity between the ESLR and the traditional SLR are even clearer: the ESLR’s OR for the likelihood of nerve root compression was 5.6 (*p* < 0.05) vs. 2.4 (*p* = 0.34) with the traditional SLR. It has already been shown that the ESLR produces reliable and repeatable results even without the knowledge of the subject’s previous medical history or other clinical findings [14], and as this knowledge is combined with our recent data, it can be debated that the ESLR is an inexpensive, diagnostically highly capable and promising clinical tool.

MRI has widely been used as the reference standard both in science and in clinical practice to confirm the results of clinical tests [2, 3, 7, 8, 10, 12] but it has been criticized for showing a high incidence of asymptomatic findings as lumbar disc herniations and various degenerative changes [5, 8, 25]. We found the same: In the control group (negative ESLR and traditional SLR), 11/20 subjects had lumbar disc herniation and 10/20 nerve root compression. These ‘false-negative’ findings were even more evident on subjects with a negative result on the traditional SLR (19/30 lumbar disc herniations and 18/30 nerve root compressions). These findings inevitably affect the test’s specificity value. Besides, even though the sensitivity values for presence of lumbar disc herniation with a positive ESLR and traditional SLR were almost the same (0.85 and 0.90, respectively), a deeper look at the cross-tabulation shows that the traditional SLR could detect only half the sciatic subjects. Following these findings, the agreement between the results of the MRI and the ESLR, measured with Cohen’s Kappa, were (not surprisingly) only fair for both lumbar disc herniation and nerve root compression, and slight with the traditional SLR. As the subjects were gathered from the patient population of a specialized spine clinic, it may have had an effect on the high incidence of the lumbar disc herniations and nerve root compressions seen in the MRIs. This raises the discussion as to whether it is more important to test for provoked symptoms instead of deriving clinical decisions from radiologic findings.

Our findings support previous cautions regarding the utilization of the MRI in clinical practice. The first problem is the high prevalence of asymptomatic abnormalities. Being nowadays more accessible for a large number of patients, MRI is frequently employed not only in an attempt to reach an accurate pathoanatomical diagnosis to a patient’s low back pain issue but also may be utilized to meet patient expectations or in an effort to alleviate the anxiety about possible underlying causes for the pain [26]. With the frequent pathologic findings linked with MRI, we still need tools to discern which MRI findings are clinically relevant and select which patients to image, particularly since changes seen in the MRI can lead to further diagnostic procedures or even surgery [3, 6–8, 26]. This need becomes even more important when low back pain often does not correlate to the structural changes seen on the MRI nor does it necessarily show new pathologic changes concurrently with the symptom aggravation [27]. Our findings with the ESLR show that it efficiently and inexpensively can discern the sciatic patients and has a high predictive value for pathology seen in the MRI when being positive, even when utilized in isolation without any knowledge of the patient’s previous history or clinical findings. Particularly, the location-specific structural differentiation movements (ankle dorsiflexion or hip internal rotation) help to analyze whether the symptoms are from the neural structures.

If/when the ESLR were to be used more as a part of the physical examination and history, its diagnostic performance may be even higher and used for better recognition of different subtypes of low back patients. As the interrater agreement of the ESLR has already been shown to be nearly perfect [14], and combined with the current data of the MRI results, ESLR seems to have high validity when positive. With the negative results, however, this seems not to be the case because MRI shows many asymptomatic ‘false-positive’ findings. Our study raises the idea that the ESLR detects a subgroup of low back pain and sciatic patients that have a functional disturbance in the nerve root - mechanical sensitivity, chemical inflammation and/or impairment of movement – that, however, may not relate contemporaneously to any specific pathology. This increased knowledge may lead to more targeted treatment options for the patients.

Our study has some limitations. The sample size was relatively small as the study group was primarily designed (in its size) to test the agreement for the results in terms of Cohen’s Kappa method following the recommendations for reliability studies in clinical sciences [16, 17]. Another limitation is that the traditional SLR was tested by a treating physician and not by blinded examiners. Our sample does not represent a true prevalence of population-wide spread of low back pain subtypes; it was adjusted to an equal number of sciatic and control patients selected from the patients sent from a primary care unit to the institutional spine center. This means it may not be entirely generalizable to a large clinical population in the primary health care setting, but then again, it represents true consecutive patients seen in a specialized spine clinic on which the ESLR shows high diagnostic performance also when performed in isolation. For the future reference, more studies with a larger population are needed to better assess ESLR performance with a characteristic distribution of low back pain patients.

We modified the well-known SLR test for two main reasons: (i) the existence of in-depth knowledge of the phenomena occurring during the test, and (ii) the possibility of providing a cost-effective tool to evaluate the patients regardless the surroundings such as MRI or other diagnostic equipment. Based on our results, the ESLR (i.e., SLR with location-specific structural differentiation) is a reliable and repeatable tool with low back patients for detecting symptoms of neural origin. Within this context it is important to emphasize, however, that the test itself cannot be utilized as a means of showing the presence of any specific pathology; rather, it may be used to specify whether the symptoms originate from somewhere along the course of the sciatic nerve or its nerve roots (for example nerve root foramina, deep gluteal space, or distal entrapments) due without determining a cause, and it is likely to exert its full potential as an integral part of the clinical examination. The ESLR may help clinicians to decide more efficiently, which patients should be referred for further evaluation to a specialist or imaging (patient selection). Ultimately, better clinical knowledge will improve the treatment of the multifaceted low back pain issues and consequently may alleviate the pressure on national healthcare systems due to unnecessary diagnostic imaging procedures.

## Conclusions

The ESLR shows high validity in detecting neural symptoms and is associated with pathology seen in the MRI when positive. However, it cannot be used as a tool to detect or exclude any specific pathologic conditions. We suggest the use of ESLR in clinical practice as an integral part of the clinical examination, where it may prove to be a valuable tool in detecting patients with sciatic (neural) symptoms, especially when combined with other clinical findings and patient history.

## Data Availability

The datasets used and/or analysed during the current study are available from the corresponding author on reasonable request.

## Abbreviations

95%CI: 95 % confidence interval
ESLR: Extended straight leg raise test
FA: Flip angle
FOV: Field of view
MRI: Magnetic resonance imaging
OR: Odds ratio
SLR: Straight leg raise test
ST: Slice thickness
TE: Echo time
TR: Repetition time
