# Correction to: Extending the straight leg raise test for improved clinical evaluation of sciatica: validity and diagnostic performance with reference to the magnetic resonance imaging

**DOI:** 10.1186/s12891-021-04781-w

**Published:** 2021-11-10

**Authors:** Janne Pesonen, Michael Shacklock, Juha-Sampo Suomalainen, Lauri Karttunen, Jussi Mäki, Olavi Airaksinen, Marinko Rade

**Affiliations:** 1 Department of Rehabilitation, Kuopio University Hospital, PL 100, 70029 KYS, Kuopio, Finland; 2 Department of Surgery (incl. Physiatry), University of Eastern Finland, Kuopio, Finland; 3 Neurodynamic Solutions, Adelaide, Australia; 4 Department of Clinical Radiology, Kuopio University Hospital, Kuopio, Finland; 5 Faculty of Medicine, University of Osijek, Orthopaedic and Rehabilitation Hospital “Martin Horvat”, Rovinj, Croatia; 6 Department of Natural and Health Studies, Juraj Dobrila University of Pula, Pula, Croatia

**Correction to: BMC Musculoskelet Disord 22, 808 (2021)**

**https://doi.org/10.1186/s12891-021-04649-z**

Following publication of the original article [1], the authors reported an error that Table 2 is missing from the publication.

The missing Table 2 is shown below. The original article [1] has been updated.

**Table 2:** Cohen's Kappa values for agreement between the MRI findings and ESLR or traditional SLR results

	Kappa	95%CI	***p***-value
**ESLR / LDH on MRI**	0.30	0.03 - 0.57	0.04
**ESLR / NC on MRI**	0.25	-0.04 - 0.54	0.10
**Trad. SLR / LDH on MRI**	0.17	-0.01 - 0.35	0.11
**Trad. SLR / LDH on MRI**	0.07	-0.16 - 0.29	0.57

*ESLR* Extended straight leg raise test, *LDH* Lumbar disc herniation, *MRI* Magnetic resonance imaging, *NC* Neural root compression, *Trad. SLR* Traditional straight leg raise test, *95%CI* 95% confidence interval
