# Correction to: Extending the straight leg raise test for improved clinical evaluation of sciatica: validity and diagnostic performance with reference to the magnetic resonance imaging

**DOI:** 10.1186/s12891-022-05315-8

**Published:** 2022-05-31

**Authors:** Janne Pesonen, Michael Shacklock, Juha-Sampo Suomalainen, Lauri Karttunen, Jussi Mäki, Olavi Airaksinen, Marinko Rade

**Affiliations:** 1 Department of Rehabilitation, Kuopio University Hospital, PL 100, 70029 KYS/Kuopio, Finland; 2 Department of Surgery (incl. Physiatry), University of Eastern Finland, Kuopio, Finland; 3 Neurodynamic Solutions, Adelaide, Australia; 4 Department of Clinical Radiology, Kuopio University Hospital, Kuopio, Finland; 5 Faculty of Medicine, University of Osijek, Orthopaedic and Rehabilitation Hospital “Martin Horvat”, Rovinj, Croatia; 6 Department of Natural and Health Studies, Juraj Dobrila University of Pula, Pula, Croatia

**Correction to: BMC Musculoskelet Disord 22, 808 (2021)**

10.1186/s12891-021-04649-z

Following publication of this article [1], the authors report the following corrections to the main text and Figs. 4 and 5:

**Fig. 4:**
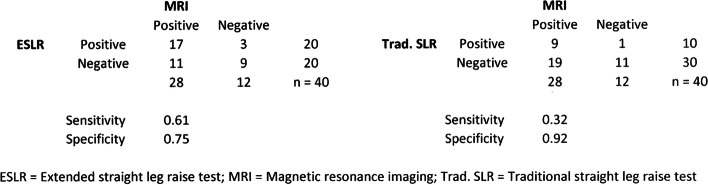
Crosstabulations between ESLR, traditional SLR and MRI findings for lumbar disc herniation. ESLR = Extended straight leg raise test; MRI = Magnetic resonance imaging; Trad. SLR = Traditional straight leg raise test

**Fig. 5:**
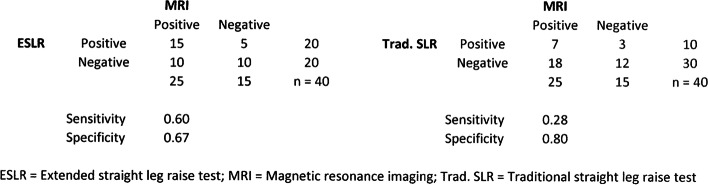
Crosstabulations between ESLR, traditional SLR and MRI findings for neural compression. ESLR = Extended straight leg raise test; MRI = Magnetic resonance imaging; Trad. SLR = Traditional straight leg raise test

i) The authors noticed that the sensitivity and specificity values for both lumbar disc herniation and nerve root compression for extended straight leg raise and traditional straight leg raise test were miscalculated due to erroneous formula in the Excel file. The reported sensitivity and specificity values have been corrected to the abstract and to the results section on Figs. 4 and 5.
  Corrected values are (old ➔ corrected):
  ESLR sensitivity for lumbar disc herniation 0.85 ➔ 0.61
  ESLR specificity for lumbar disc herniation 0.45 ➔ 0.75
  Traditional SLR sensitivity for lumbar disc herniation 0.90 ➔ 0.32
  Traditional SLR specificity for lumbar disc herniation 0.37 ➔ 0.92 (all Fig. 4).
  ESLR sensitivity for nerve root compression 0.75 ➔ 0.60
  ESLR specificity for nerve root compression 0.50 ➔ 0.67
  Traditional SLR sensitivity for nerve root compression 0.70 ➔ 0.28
  Traditional SLR specificity for nerve root compression 0.40 ➔ 0.80 (all Fig. 5).

ii) With the corrected values to the sensitivity and specificity, two sentences in the Discussion’s third paragraph had minor changes. The focus of the study, main discussion or the message of the study remains unchanged and unaffected. Also, the conclusion of the study remains unchanged.
